# The Role of Antibody-Based Therapies in Neuro-Oncology

**DOI:** 10.3390/antib12040074

**Published:** 2023-11-13

**Authors:** Rishab Ramapriyan, Jing Sun, Annabel Curry, Leland G. Richardson, Tarun Ramesh, Matthew A. Gaffey, Patrick C. Gedeon, Elizabeth R. Gerstner, William T. Curry, Bryan D. Choi

**Affiliations:** 1 Department of Neurosurgery, Massachusetts General Hospital, Boston, MA 02114, USAacurry25@andover.edu (A.C.); lrichardson7@mgh.harvard.edu (L.G.R.); wcurry@mgh.harvard.edu (W.T.C.); 2 Harvard Medical School, Boston, MA 02115, USAegerstner@mgh.harvard.edu (E.R.G.); 3 Department of Surgery, Brigham and Women’s Hospital, Boston, MA 02115, USA; 4 Department of Neurology, Massachusetts General Hospital, Boston, MA 02114, USA

**Keywords:** immunotherapy, brain tumor, antibodies, immunomodulation, glioblastoma, meningioma

## Abstract

This review explores the evolving landscape of antibody-based therapies in neuro-oncology, in particular, immune checkpoint inhibitors and immunomodulatory antibodies. We discuss their mechanisms of action, blood-brain barrier (BBB) penetration, and experience in neuro-oncological conditions. Evidence from recent trials indicates that while these therapies can modulate the tumor immune microenvironment, their clinical benefits remain uncertain, largely due to challenges with BBB penetration and tumor-derived immunosuppression. This review also examines emerging targets such as TIGIT and LAG3, the potential of antibodies in modulating the myeloid compartment, and tumor-specific targets for monoclonal antibody therapy. We further delve into advanced strategies such as antibody–drug conjugates and bispecific T cell engagers. Lastly, we explore innovative techniques being investigated to enhance antibody delivery, including CAR T cell therapy. Despite current limitations, these therapies hold significant therapeutic potential for neuro-oncology. Future research should focus on optimizing antibody delivery to the CNS, identifying novel biological targets, and discovering combination therapies to address the hostile tumor microenvironment.

## 1. Background

Neuro-oncology is a rapidly evolving field focusing on the management of primary and secondary brain and spinal cord tumors. The American Cancer Society predicts that 24,810 adults in the US (14,280 males and 10,530 females) will be diagnosed with primary malignant tumors of the brain and spinal cord in 2023 [1]. Around 18,990 fatalities (11,020 males and 7970 females) due to primary malignant brain and central nervous system (CNS) tumors, the tenth leading cause of death for both genders, are expected to occur in the US in 2023 [1]. Given the high mortality rate of patients with neuro-oncological conditions, new therapeutic approaches are needed to improve the standard of care.

Antibody-based immunotherapy holds great potential for patients with CNS malignancies, with a myriad ongoing preclinical and clinical efforts. With advances in producing humanized antibodies and “fully-human” proteins [2], antibodies have been shown to be potent therapeutic tools in the treatment of various cancers starting from the late 1980s [3,4]. However, the CNS presents unique challenges for antibody therapies, in large part due to the immunosuppressive microenvironment [5] and limited access hampered by the blood-brain barrier (BBB) [6].

This review aims to discuss the role of antibody-based therapies in neuro-oncology, focusing on immune checkpoint inhibitors and immunomodulatory antibodies. We will explore their mechanisms of action, their ability to penetrate the BBB, and their efficacy in treating neuro-oncological conditions.

## 2. The Challenge of Crossing the BBB

The BBB, a highly selective semipermeable structure, restricts the passage of substances from the bloodstream into the brain. This barrier poses a significant challenge for the delivery of antibody-based therapeutic agents [7]. Monoclonal antibodies are proteins that are traditionally considered to be too large to penetrate the blood-brain barrier (BBB), limiting their efficacy against tumors within the CNS [8]. Additionally, the expression of the neonatal Fc receptor (FcRn) in the capillary endothelium of the BBB has a putative role in preventing the delivery of antibodies to the brain parenchyma. Specifically, FcRn is theorized to cause reverse transcytosis of IgG antibodies from the brain to the blood [9,10]. Modifying the antibody Fc domain to prevent their interaction with FcRn has been shown preclinically to improve the distribution of antibodies to the brain [9,11].

However, the traditional limitations of the BBB may be less relevant to brain tumors that disrupt the integrity of the BBB. In the case of high-grade gliomas, the accumulation of contrast agents like gadolinium and a higher distribution of high molecular weight proteins (i.e., monoclonal antibodies) within tumor tissue compared to normal brain tissue suggests that the compromised barrier may permit the passage of molecules and/or biologics to the brain from systemic circulation [12,13,14]. Although a more permeable BBB is a hallmark of high-grade glioma and perhaps beneficial for drug delivery, it is thought that much of the infiltrative component may still be protected by intact regions of the BBB [12], representing a persistent challenge for antibody-based therapeutics. Both the timing of antibody administration and the extent of BBB permeability in neuro-oncologic disease are areas of ongoing research.

Evidence from other neurological antibody-based therapies and autoimmune neurological diseases suggest some BBB penetration potential for monoclonal antibodies. In mouse studies of Alzheimer’s disease, peripherally administered antibodies against amyloid-beta were able to enter the CNS, localize to plaques, and clear them [15]. This suggests some degree of BBB crossing, but theoretically, the antibodies may have accumulated outside the BBB and sequestered plaques there. In paraneoplastic neurologic disorders, several antibodies that are generated peripherally against a tumor can cross-react with neurological structures and cause morbidity. This in turn suggests that these paraneoplastic antibodies such as anti-Ma, anti-Ri, and anti-amphiphysin can cross the BBB and bind the brain parenchyma [16].

Finally, clinical studies using radiolabeled antibody–drug conjugates have shown selective tumor localization of these antibodies in patients with gliomas and other intracranial malignancies, providing further credence to the ability of monoclonal antibodies to accumulate in intracerebral sites [17,18]. However, rigorous comparison of intratumoral versus systemic delivery of therapeutic antibodies is ongoing, and radiolabeling of other frequently infused monoclonal antibodies like immune checkpoint inhibitors has not been studied comprehensively in patients with brain tumors. Recently, bispecific antibodies that bind to transferrin receptors (TfR) associated with the BBB have been shown to have enhanced distribution to the brain through co-opting the mechanism of TfR-mediated endocytosis [19]. Bispecific antibodies targeting other components of the BBB may prove effective in facilitating the delivery of therapeutic agents to the brain.

Even if these barriers posed by the BBB prevent monoclonal antibodies from entering the brain, there remains an open question on whether immunomodulatory antibodies, such as the immune checkpoint inhibitors that we will discuss in the next section, need to reach the brain to have efficacy. Antibodies could theoretically bind to and modulate T cells in the periphery, such that these cells are activated in some way prior to homing to the CNS for their cytotoxic activities.

## 3. Immune Checkpoint Inhibitors and Immunomodulatory Antibodies

### 3.1. Immune Checkpoint Inhibitors: Anti-PD-1 and Anti-CTLA-4

Immune checkpoint inhibitors (ICIs), such as anti-programmed cell death protein 1 (PD-1) and anti-cytotoxic T-lymphocyte-antigen 4 (CTLA-4), have revolutionized cancer treatment by augmenting the immune system’s ability to destroy cancer cells through the activation and prevention of T cell exhaustion. ICIs are not involved in tumor recognition but rather target the tumor–immune cell interface. Specifically, these inhibitors work by blocking the signals that cancer cells use to evade immune responses, thereby allowing immune cells to attack tumors.

PD-1 is a cell surface receptor expressed predominantly on activated T cells, B cells, and some monocytes. It plays a crucial role in downregulating the immune system and promoting self-tolerance by suppressing T cell inflammatory activity. The mechanism of action of PD-1 revolves around its interaction with its ligands, PD-L1 and PD-L2. When these ligands, often expressed on tumor cells or on cells in the tumor microenvironment, bind to PD-1, they transmit an inhibitory signal into the T cell [20]. This signal reduces the proliferation of T cells in lymph nodes and their activity in peripheral tissues, resulting in reduced autoimmunity and protection of tissues from immune-mediated damage. However, cancers exploit this pathway, using PD-L1 and PD-L2 expression to shut down the immune response against them, thereby allowing them to grow unchecked. One strategy for antibody-based immune checkpoint therapies is the use of anti-PD-1 antibodies (e.g., nivolumab and pembrolizumab) [21]. Therapeutic antibodies targeting the PD-1/PD-L1 pathway are designed to block this interaction, thereby releasing the “brakes” on the immune system and enabling it to target and destroy tumor cells. The function of these antibodies is to block the binding of PDL-1 to PD-1 on the surface of T cells. Use of PD-1 blockade on PD-1 expressing T cells has been associated with reversing T-cell exhaustion and improved cytokine production [22].

CTLA-4 is another critical immune checkpoint receptor, predominantly expressed on the surface of T cells. It shares structural similarities with the T cell co-stimulatory protein, CD28, and both bind to CD80 and CD86 on antigen-presenting cells (APCs). The binding of CD28 to CD80 and CD86, along with the binding of the T cell receptor (TCR) to MHC1, promotes T cell activation and proliferation [23]. However, unlike CD28, which promotes T cell activation when engaged, CTLA-4 acts as an “immune brake” by outcompeting CD28 for its ligands and delivering inhibitory signals to the T cell. In essence, CTLA-4′s engagement reduces T cell proliferation, IL-2 production, and cell cycle progression [23]. Tumor cells can exploit the CTLA-4 pathway, indirectly facilitating a suppressed immune environment favorable for tumor growth. Monoclonal antibodies, such as ipilimumab, bind to CTLA-4 to block its interaction with ligands, CD80 and CD86, thereby augmenting T cell activation and proliferation. Furthermore, CTLA-4 signaling in regulatory T cells (Tregs) promotes their suppressive function, contributing to peripheral tolerance. While some studies have previously shown that anti-CTLA-4 monoclonal antibodies selectively depleted intratumoral FOXP3^+^ regulatory T cells via an Fc-dependent mechanism, clinical evidence has not confirmed this mechanism of action [24]. In neuro-oncology, these ICIs have shown promise for a subset of patients with glioblastoma (GBM), the most common and aggressive primary brain tumor, suggesting that certain biomarkers may be good for stratifying therapies [25]. Investigators have delved into the potential biomarkers associated with ICI responsiveness in GBM patients [26]. These include relationships with the differentiation status of tumor-infiltrating lymphocytes and the role of cytotoxic CD8^+^ tumor-infiltrating lymphocytes (TILs) [27]. For example, Eomes^hi^T-bet^lo^ CD8^+^ TILs were associated with lower T cell activation in the setting of ICIs [27]. Additionally, higher densities of proliferating CD8^+^ T cells and a higher ratio of CD8^+^ to CD4^+^ cells in tumor infiltrates were found to be associated with improved survival [28].

Anti-PD-1 therapies are also currently being actively investigated in meningiomas. Preclinical evidence suggests that there is great intra- and intertumor heterogeneity of PD-L1 expression in meningiomas [29]. PD-L1 expression was also shown as a tumor marker that can predict recurrence [29]. Thus, a number of clinical trials have been conducted to evaluate the efficacy of anti-PD-1 therapy in recurrent meningiomas, as we will discuss below [30].

Although ICIs offer a promising avenue for glioma and meningioma treatment, their efficacy may be influenced by a range of factors, including the unique immune environment of the CNS, the presence and differentiation status of TILs, and the potential for synergistic combination therapies. Additional research is essential to fully realize the potential of ICIs in brain tumor treatment.

### 3.2. Combination Treatment Strategies

ICIs may be best used alongside conventional treatments like radiotherapy and chemotherapy, as well as novel modalities like virotherapy [31]. Combining virotherapy with immunotherapy may offer a novel approach to GBM in particular. Saha et al., investigated the combination of oncolytic herpes simplex virus (oHSV) with ICIs [32]. Their findings suggest that a specific oHSV expressing IL-12, when combined with anti-PD-1 and anti-CTLA-4 checkpoint inhibitors, could be curative. This combination therapy was found to be particularly effective due to the involvement of CD4^+^ and CD8^+^ T cells, as well as macrophages, creating a complex but effective interplay in combating tumors.

Radiation, which is intrinsically immunogenic and can reprogram an immunosuppressive tumor microenvironment [33], may be an effective combination therapy to facilitate immune cell recruitment and subsequent priming. Radiation has been shown to cause dynamic alterations in tumor-associated macrophages and microglia in glioma, but the extent of implications for immunotherapy still are unclear [34]. One preclinical study showed that the combination of an anti-PD-1 blockade and localized radiotherapy improved long-term survival in mice with intracranial glioma [35].

### 3.3. Clinical Trials for Anti-PD-1 and Anti-CTLA-4 Therapies

Over the past decade, a variety of trials have been conducted for ICIs in GBM [36,37,38,39,40,41,42,43,44,45]. In some of these trials, there was evidence of tumor immune microenvironment changes such as enhanced expression of chemokine transcripts, increased immune cell infiltration, and expanded T cell clonal diversity, suggesting that that antibody-based therapy can mediate immunomodulatory effects for tumors in the brain [45]. The majority of these trials utilized intravenous delivery, which raises the question regarding whether adequate BBB penetration was achieved or whether CNS localization was necessary for biological activity. One phase I trial examined intracerebral administration of CTLA-4 and a PD-1 immune checkpoint blockade in recurrent GBM [38]. While there was encouraging improvement in overall survival compared to a historical cohort, no significant effect was observed for progression-free survival. Nivolumab failed to show efficacy in the large randomized, multi-centered phase 3 Checkmate 143 trial, with comparable overall survival to bevacizumab [37]. Interestingly, the neoadjuvant administration of anti-PD-1 in an early-phase randomized trial was more effective in the setting of recurrent GBM compared to the adjuvant group [39].

Most recently, a phase 2 study of pembrolizumab in patients with recurrent and residual high-grade meningiomas achieved a 6-month progression-free survival rate of 0.48 and had a median progression-free survival (PFS) of 7.6 months. [46]. However, one in five patients experienced at least one grade 3 or higher treatment-related adverse event. Another phase 2 trial of nivolumab in patients with recurrent meningioma found no clinical benefit overall, but the drug was well tolerated [47]. The baseline TIL density in these patients was low, but three patients had increased TIL density post-treatment, and two of the three patients had an elevated tumor mutational burden greater than 10/Mb. Tumor mutational burden has been associated with positive immunotherapy responses in other tumors [48].

A phase 1/2 trial examining oncolytic virotherapy plus pembrolizumab in recurrent GBM was also recently completed [49]. In this trial, patients with GBM received intratumoral delivery of virus DNX-2401, followed by intravenous pembrolizumab. The objective response rate was 10.4% and the overall survival rate at 12 months was 52.7%. Notably, 3 of the 49 patients in the trial had durable responses and remained alive at 60 months. Genomic and immunological analysis of these tumors suggest that immune cell infiltration and checkpoint expression may be predictors of response to treatment.

The endpoint results of key clinical trials of ICIs in GBM and meningioma are summarized in Table 1.

### 3.4. Additional Immune Checkpoints: TIGIT and LAG3

T cell immunoreceptor with Ig and ITIM domains (TIGIT) and Lymphocyte-activation gene 3 (LAG3) are other immune checkpoints that have been targeted for cancer treatment [50,51]. These checkpoints are expressed on various immune cells, including T cells and natural killer (NK) cells, and their blockade can enhance immune responses against cancer cells. While research on these targets in neuro-oncology is still in its early stages, preliminary studies suggest potential therapeutic benefits. For instance, the co-blockade of PD-1 and TIGIT has been shown to enhance T cell responses in preclinical models of GBM, leading to reduced tumor growth and improved survival [52]. Additionally, TIGIT expression on TILs in patient-derived GBM samples has been found to be elevated. LAG-3 blockade via monoclonal antibodies alone or in combination with anti-PD-1 has been shown to eradicate GBM tumors in mice [53]. Clinical trials testing antibodies against TIGIT (NCT04656535) and LAG3 (NCT02658981) are currently ongoing.

### 3.5. Modulating the Myeloid Compartment with Antibodies

Most antibody-directed targets in gliomas are focused on T cell modulation, despite a growing understating of the crucial role of innate immunity and the need to redirect predominant immunosuppressive myeloid cell populations in malignant gliomas [54]. Myeloid-derived suppressor cells (MDSCs), along with immunosuppressive M2 tumor-associated macrophages, can significantly blunt the efficacy of natural and engineered anti-tumor immunity.

Distinct from immune checkpoint molecules, phosphatidylserine (PS) is an immunosuppressive target widely expressed on the surface of cancer cells, including GBM [55]. Blocking PS signaling with anti-PS monoclonal antibodies (e.g., bavituximab) has demonstrated durable anti-tumor responses, especially in combination with RT in various preclinical tumor models [56], including GBM [57]. Anti-PS therapy has been shown to repolarize the immunosuppressive myeloid compartment towards a pro-inflammatory state [56,58]. A recently published phase 2 single-arm trial evaluated the efficacy of bavituximab in combination with temozolomide and radiation in 33 newly diagnosed patients with GBM and showed a marked reduction in infiltrating myeloid-derived suppressor cells (MDSCs) after treatment [59]. Although this study demonstrates a potential on-target effect, the mechanisms by which PS targeting antibodies interfere with the inhibitory signaling and function of the myeloid compartment are not well understood and require further investigation.

The CD47 protein in lay terms is a “don’t eat me signal” that inhibits macrophage phagocytosis of tumor cells which overexpress this protein. Researchers have also investigated using anti-CD47 monoclonal antibodies to reprogram the tumor-associated macrophages in the microenvironment. Testing this therapy on human glioblastoma cell lines and xenografts, they showed that the addition of anti-CD47 led to increased tumor-cell phagocytosis in both M1 and M2 macrophages, but this increase was more prominent in M1 macrophages [60]. Anti-CD47 therapy has also led to durable responses in various preclinical pediatric brain tumor models [61]. However, the trials of anti-CD47, which were initially tested in hematological malignancies were discontinued due adverse effects of significant anemia, which may suggest off-target toxicity because CD47 is expressed on non-malignant blood cells. Further study and optimization of the anti-CD47 may allow the optimization of the therapy.

### 3.6. Neurotoxicity Profile of Immune Checkpoint and Modulatory Antibodies

The use of antibody therapies has the potential to mediate off-tumor, on-target effects [62,63,64]. These immune-related adverse effects (irAEs) can include colitis, thyroiditis, pneumonitis, rashes, hepatitis, and less commonly, neurologic syndromes. Proposed mechanisms include overactivation of the peripheral immune system, activation from viral or co-administered drug antigens, and failure of self-tolerance [65,66]. Multiple case reports have described neurologic irAEs (irAEs-N), and previous research has found that up to 4.2% of cancer patients treated with anti-PD-1 therapy exhibited some level of neurologic dysfunction, with most occurring within 3 months of starting therapy [67]. Other studies estimate 1–2% will have irAEs-N [68]. Combination therapies can increase this likelihood up to a 12% risk [69]. The heterogeneity of irAEs-N within this population is notable for the involvement of both the central and peripheral nervous system, with syndromes including headaches, seizures, peripheral neuropathy, demyelinating diseases, neuromuscular receptor dysfunction, diffuse encephalopathy, CNS vasculitis, and aseptic meningitis [70,71,72].

For patients with pre-existing immune-related neurologic diseases (e.g., myasthenia gravis), exacerbation of the host immune response can be fatal [70]. The distribution of severity influence grading on the Common Terminology Criteria for Adverse Events (CTCAE) ranges from grade 1 (representing asymptomatic and mild disease) to grade 3 (requiring hospitalization) and grade 5 (mortality) [73]. For grade 3 and higher, ASCO (2021) and NCNN (2022) guidelines recommend hospitalization and treatment with intravenous glucocorticoids and additional appropriate immunomodulating/immunosuppressive therapies including intravenous immunoglobulin (IVIG), CD20 inhibition, and plasmapheresis [74,75,76]. After discontinuation of immunotherapy and treatment with immunosuppression, the majority of patients have robust clinical improvement, although residual sequalae may persist [77]. Balancing efficacy with toxicity remains a critical component of immunotherapy, and further research into the prediction and effective management of irAEs-N could improve tumor response to therapy. The pattern of adverse events in ICI neuro-oncology trials conducted to date has not been significantly different from that of other types of cancer. However, most neuro-oncology trials have utilized intravenous delivery. One trial for GBM which used intracerebral ICI delivery reported that immune-related adverse events were mild and infrequent [38]. It will be important to further characterize the neurotoxicity risk associated with intracerebral delivery.

If immune checkpoint blockade therapy is concurrently used with chimeric antigen receptor (CAR) T cell delivery, additional neurotoxic effects including cytokine release syndrome (CRS), immune effector cell-associated neurotoxicity syndrome (ICANS), and tumor inflammation-associated neurotoxicity (TIAN) should be considered [78,79,80]. CRS is defined by a fever and nonspecific symptoms, including fatigue, tachycardia, and a rash, which may be self-limiting or require additional critical care interventions [81,82]. Patients with ICANS develop neurologic dysfunction with characteristic aphasia, apraxia, and dysgraphia which can progress to encephalopathy [83,84]. The Immune Effector Cell Encephalopathy Score (ICE) characterizes the severity, informing appropriate interventions for ICANS [85].

## 4. Tumor-Specific Targets for Monoclonal Antibody-Based Therapy

Reaching optimal treatment outcomes in brain tumors largely depends on the appropriate selection of targets. Some frequently explored surface targets in the field of neuro-oncology are discussed below.

### 4.1. EGFRvIII

This is a variant of the Epidermal Growth Factor Receptor (EGFR), resulting from a deletion of exons 2 to 7 [86,87]. In GBM, nearly 40% of newly identified patients show EGFR gene amplification, with roughly half of those expressing EGFRvIII [88]. The modified extracellular domain structure brought about by this mutation provides an ideal tumor-specific target due to its unique epitope, which may be recognized by specific monoclonal antibodies [89], reducing the chance of unwanted toxicity. EGFRvIII-targeted vaccination therapy using a peptide vaccine has been successful in redirecting cellular and humoral immunity against this target in cancer cells, preclinically and clinically [90,91,92].

### 4.2. IL13Rα2

IL-13′s function is to manage inflammation and immune responses upon binding to IL13Rα1. Furthermore, IL-13 also attaches to IL13Rα2 with high affinity. IL13Rα2 is found in more than 75% of GBMs and is associated with tumor aggressiveness and poor prognosis [93,94,95]. However, it is not significantly present in normal brain tissue or most other normal tissues, except for the testes [96]. Given the relatively specific presence of IL13Rα2 in GBM, it may serve as a promising target.

### 4.3. HER2

Human Epidermal Growth Factor Receptor 2 (HER2), a type of receptor tyrosine kinase, is overexpressed in around 40% of medulloblastomas and 80% of GBMs [81,97], with no expression in normal brain tissues. Thus, it appears to be an ideal target antigen for antibody-based immunotherapy in neuro-oncology. However, the use of HER2-targeted immunotherapy carries a risk of harmful off-target effects, as HER2 is also found in several essential normal tissues [98]. Targeting HER2 with chimeric antigen receptor (CAR) T cells has led to severe immune toxicity and subsequent multi-organ system failure in clinical studies [99].

### 4.4. Disialoganglioside (GD2)

GD2 exhibits high expression in several cancers including neuroblastoma and GBMs, making it an attractive target for GBM therapy as it is present on GBM cell lines and patient samples [100]. GD2 has been effectively and safely targeted by CAR T cells in diffuse midline gliomas and neuroblastoma in recently published clinical trials [101,102].

### 4.5. CD147

CD147 is found in GBM and is associated with a poorer prognosis for patients [103]. It plays a role in the degradation of the extracellular matrix (ECM) and promotes tumor progression, invasion, and metastasis.

### 4.6. SMARCAL1 Mutations

A certain group of primary GBMs which carry mutations in the SWI/SNF-related matrix-associated actin-dependent regulator of the chromatin subfamily A-like protein 1 (SMARCAL1) are seen as a potential target [104].

### 4.7. Delta-Like Canonical Notch Ligand 3 (DLL3)

DLL3, part of the Delta/Serrate/Lag2 (DSL) Notch receptor ligand family, plays a role in Notch signaling, impacting various cell processes such as differentiation, proliferation, survival, and apoptosis. Cell surface DLL3 may represent an effective target for certain subsets of gliomas and is expressed at very low or absent levels in normal tissues [105,106]. Rovalpituzumab tesirine (Rova-T) is a DLL3-targeting antibody–drug conjugate, and Tarlatamab [107], a first-of-its-kind DLL3-targeted bispecific T Cell engager, was recently studied in a phase I trial for recurrent small-cell lung cancer patients [108].

## 5. Advanced Antibody-Based Strategies

### 5.1. Antibody–Drug Conjugates (ADCs)

Antibody–drug conjugates consist of a monoclonal antibody fused with a potent cytotoxic agent. These cytotoxic components fall into two categories: immunotoxins and radiolabeled antibodies. Immunotoxins represent antibodies that are linked to naturally derived bacterial toxins, such as exotoxin A from Pseudomonas aeruginosa or diphtheria toxin. Some examples of these are ABT-414 [109], AMG-595 [110], Cintredexin Besudotoxin [111], NBI-3001 [112], TP-38 (IVAX) [113], and Tf-CRM107 [114].

Radiolabeled antibodies, also known as radioimmunoconjugates, are monoclonal antibodies connected to a radionuclide. These radiolabeled antibodies employ isotopes like iodine-124 or iodine-131 as their toxic payloads. Compared to traditional radiation therapy, radioimmunotherapy (RIT) reduces toxicity and boosts the effectiveness of monoclonal antibodies. Several radionuclides, including actinium-225 (225 Ac), astatine-211 (211 At), bismuth-213 (213 Bi), indium-111 (111 In), iodine-123 (123 I), iodine-124 (124 I), iodine-131 (131 I), lead-212 (212 Pb), lutetium-177 (177 Lu), technetium-99 m (99 mTc), copper-64 (64 Cu), gallium-68 (68 Ga), yttrium-86 (86 Y), yttrium-90 (90 Y), and zirconium-89 (89 Zr), can be used for labeling in therapeutic applications [115].

In the late 1980s, Bigner and colleagues began investigating iodine-131-labeled antibodies against tenascin, a glioma-associated extracellular matrix antigen, in intracranial human xenografts [116]. The preclinical studies showed significant survival prolongation in mice bearing these tumors. Later, Bigner and Pastan led studies on using Pseudomonas exotoxin conjugated to antibodies targeting IL-4, EGFR, and EGFRvIII which were translated to phase 1 trials using intratumoral administration [117]. Despite the impressive potency of these antibody–drug conjugates, early clinical trials showed limited success and some notable toxicity [118], especially with EGFR-targeting ADCs [119]. More recently, ABT-806 (Depatuxizumab), an ADC against EGFRvIII, failed to show any survival benefit in a phase III clinical trial [120]. Thus, the therapeutic potential of ADCs in clinical settings remains to be clinically realized, but the optimization of ADC targets and payloads, along with combinatorial strategies, continues to be explored.

### 5.2. Bispecific T Cell Engagers (BiTEs)

BiTE antibodies typically consist of one scFv that specifically identifies a surface antigen on a target cell, and another scFv that recognizes CD3, a portion of the activating complex found on T cells. BiTEs have demonstrated the ability to redirect T cells in the treatment of hematologic malignancies [121]. In preclinical studies, a BiTE that targets EGFRvIII has been observed to stimulate T cells and facilitate the antigen-specific destruction of EGFRvIII-expressing gliomas [122]. BiTEs have also been shown to be potent in modifying the tumor microenvironment by converting and redirecting the regulatory T cells into anti-tumor effector cells [123]. One theorized mechanism of how BiTEs reach the brain is that they piggyback on circulating T cells that then traffic to the brain parenchyma [124]. In support of this hypothesis, data show that the adoptive transfer of activated T cells increases BiTE CNS tumor penetrance [125]. This may represent a novel mechanism for enhancing antibody delivery to the brain through the hijacking of T cell migration.

### 5.3. Using Adoptive Cell Therapy to Enhance Antibody Delivery to the CNS

As aforementioned, antibody-based therapies for brain tumors are often systemically administered, and their therapeutic success may often hinge on their ability to cross the BBB or act on target cells that can ultimately traffic past the endothelial wall of the BBB [8]. Designing a strategy to optimize antibody-based delivery in gliomas is quite challenging; however, several novel approaches are currently being investigated, such as receptor mediated-transcytosis, focused ultrasound, nanoparticles, chemical modifications, and enzyme-mediated BBB disruption [126,127,128].

Another method to enhance antibody delivery in a controlled manner includes utilizing adoptive cell transfer (ACT) [129]. CAR T cells can be modified to possess the ability to secrete proteins (i.e., antibodies, cytokines, chemokines, or enzymes) and act as living “micro-pharmacies” [130]. A key benefit of this strategy is exploiting the T cell’s ability to successfully traverse the BBB, traffic to tumor sites, and deliver a payload. Moreover, the localized delivery of antibodies in the TME has been shown to lower the likelihood of toxicities associated with systemic infusion [131]. For example, localized delivery of a bispecific T cell engager (BiTE) specific for wild-type EGFR by an EGFRvIII-directed CAR T cell in GBM prevented on-target toxicity in normal tissues [132]. Engineering T cells to secrete other immunomodulating antibody-based molecules locally in the glioma TME is an attractive approach that offers the potential for versatility along with safety.

## 6. Conclusions

**Figure 1:**
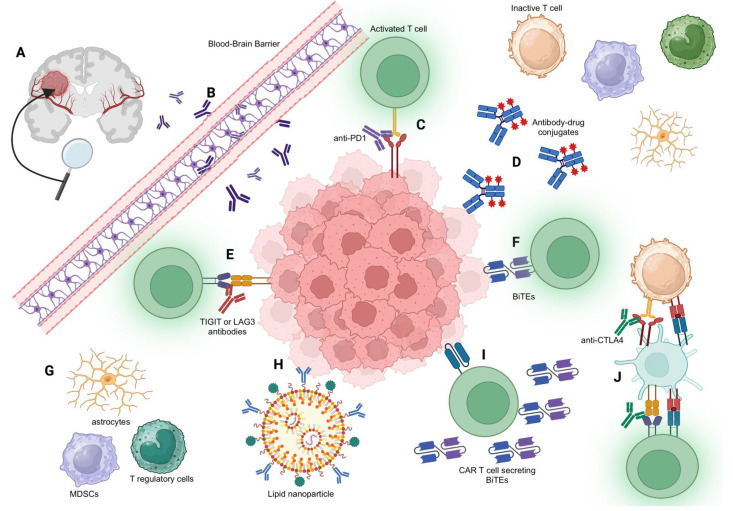
Illustration of Antibody Mechanisms in Neuro-oncology. (**A**) Overview of a centrally located brain tumor, with its vascular supply highlighted. (**B**) Detailed view of antibodies attempting to cross the blood-brain barrier, emphasizing the inherent challenges of this process. (**C**) PD-1 antibodies in action, illustrating their role in enhancing T cell functionality against tumor cells. (**D**) Depiction of antibody–drug conjugates (ADCs) that are attached to a cytotoxic drug, targeting and destroying tumor cells. (**E**) TIGIT or LAG-3 antibodies activating T cells, emphasizing their function in boosting immune response against tumor cells. (**F**) BiTE (Bispecific T cell Engager) mechanism, showcasing how it links T cells to tumor cells, facilitating the destruction of the tumor cells by T cells. (**G**) Illustration of other cells within the tumor microenvironment (TME) such as astrocytes, monocytes, and myeloid-derived suppressor cells (MDSCs). These cells can exert immunosuppressive effects, hindering effective anti-tumor immune responses. (**H**) Representation of lipid nanoparticles carrying antibody receptors, illustrating the potential for targeted drug delivery. (**I**) CAR T cell mechanism depicted as it releases BiTEs upon activation, further enhancing T cell engagement with tumor cells. (**J**) Detailed depiction of the anti-CTLA4 antibody mechanism: The anti-CTLA4 antibody blocks the interaction between CTLA-4 on both T regulatory and T effector cells and B7 on dendritic cells. In effector cells, CTLA-4 acts as an inhibitory receptor, reducing T cell activation. By blocking this interaction, the anti-CTLA4 antibody promotes prolonged T cell activation and increased T effector cell activity against tumor cells.

In conclusion, antibody-based therapies represent a promising frontier in neuro-oncology (Figure 1). ICIs, despite their current limitations, show potential in modulating immune microenvironments in the CNS. Ultimately, the success of antibody-based therapies will depend on overcoming both the challenges posed by the BBB and the immunosuppressive TME. Various strategies to enhance delivery into the CNS, such as receptor-mediated transcytosis, focused ultrasound, and nanoparticles, are under active investigation and should be explored in combination with antibody-based therapy for brain tumors. There also remain fundamental questions regarding the immune dynamics of the brain, which may influence antibody-delivery strategies. For example, it is unclear whether ICIs need to penetrate the BBB to have efficacy or if they can peripherally activate T cells that then traffic to the brain. The location of T cell priming and activation with relation to brain tumors, which has not been fully elucidated, may also influence the choice between delivering immunomodulatory antibodies locally (e.g., intratumoral or intrathecal injection) versus peripherally. Combination therapy of immune checkpoint inhibitors with radiotherapy and virotherapy, as well as combinatorial antibody strategies to target both T cells and the TME in tandem, are promising. Questions related to the optimal sequencing and specific combinations of such therapies merit further study. Continued research and innovation are essential to unlock the full potential of these promising therapeutic avenues in neuro-oncology.

## Figures and Tables

**Table 1:** Key trials of immune checkpoint inhibitors for central nervous system tumors.

Author	Year	Phase	Tumor Type	Immunotherapy Agent	Combination Treatment	Number of Patients	Progression-Free Survival	Overall Survival
Brastianos PK, et al. [46]	2022	Phase II	recurrent high-grade meningioma	Pembrolizumab	None	25	7.6 months (90% CI, 3.4–12.9)	20.2 months (90% CI, 14.8–25.8)
Nassiri F, et al. [49]	2023	Phase I/II	recurrent glioblastoma	Pembrolizumab	Oncolytic Virotherapy	49	Not reported	12.5 months (10.7–13.5)
Reardon DA, et al. [36]	2021	Phase I	recurrent glioblastoma	Pembrolizumab	None	111	2.8 months (95% Cl, 1.8–8.1)	13.1 months (95% CI 8.0–26.6)
Reardon DA, et al. [37]	2020	Phase III	recurrent glioblastoma	Nivolumab	Ipilimumab	369	1.5 months (95% CI, 1.5–1.6)	9.8 months (95% CI, 8.2–11.8)
Duerinck J, et al. [38]	2021	Phase I	recurrent glioblastoma	Ipilimumab	Nivolumab	27	2.9 months (95% CI, 2.5–3)	9.5 months (95% CI: 6.8–12.3)
Cloughesy TF, et al. [39]	2019	Phase II	recurrent glioblastoma	Pembrolizumab	None	35	3.3 months	13.7 months
Nayak L, et al. [40]	2021	Phase II	recurrent glioblastoma	Pembrolizumab	Bevacizumab	80	4.1 months (95% CI, 2.8–8.6)	8.8 months (95% CI, 7.7–14.2)
Sahebjam S, et al. [41]	2020	Phase I	recurrent high-grade glioma	Pembrolizumab	Hypofractionated Stereotactic Irradiation	32	7.9 months (95% CI, 6.3–12.5)	13.34 months (95% CI, 9.5–18.5)
Bi WL, et al. [47]	2022	Phase II	recurrent high-grade meningioma	Nivolumab	None	25	5.6 months (95% CI, 3.2–7.4)	30.9 months (95% CI, 17.6, NA)
Omuro A, et al. [42]	2023	Phase III	newly diagnosed glioblastoma	Nivolumab	Radiation Therapy	560	6.0 months (95% CI, 5.7–6.2)	13.4 (95% CI, 1.1–1.6)
Lim M, et al. [43]	2022	Phase III	newly diagnosed glioblastoma	Nivolumab	Temozolomide Plus Radiation Therapy	716	10.6 months (95% CI, 8.9–11.8)	28.9 months (95% CI, 24.4–31.6)
Omuro A, et al. [44]	2018	Phase I	recurrent glioblastoma	Nivolumab	Ipilimumab	40	1.5 months (95% CI, 0.5–2.8)	9.2 months (95% CI, 3.9–12.7)
Schalper KA, et al. [45]	2019	Phase II	recurrent glioblastoma	Nivolumab	None	30	4.1 months (95% CI, 2.8–5.5)	7.3 months (95% CI, 5.4–7.9)
